# A decade of *GigaScience*: 10 years of the evolving genomic and biomedical standards landscape

**DOI:** 10.1093/gigascience/giac047

**Published:** 2022-05-17

**Authors:** Lynn M Schriml

**Affiliations:** University of Maryland School of Medicine, Institute for Genome Sciences, Baltimore, MD 21201, USA

## Abstract

Standardization of omics data drives FAIR data practices through community-built genomic data standards and biomedical ontologies. Use of standards has progressed from a foreign concept to a sought-after solution, moving from efforts to coordinate data within individual research projects to research communities coming together to identify solutions to common challenges. Today we are seeing the benefits of this multidecade groundswell to coordinate, exchange, and reuse data; to compare data across studies; and to integrate data across previously siloed resources.

## Background

Looking back a decade or more, the heyday of collecting and reporting genomic and biomedical data was primarily driven by project-by-project needs rather than the needs of multiple projects or entire fields. Over time, however, the sheer depth of data demanded solutions to make data FAIR: Findable, Accessible, Interoperable, and Reusable. Even before FAIR became the mantra of the day, individual researchers and research consortia initiated the development of clinical vocabularies, thesauri, and minimal information standards to maximize the utility of the data produced with the intention to future-proof data for purposes yet to come. In the clinical vocabulary namespace, the World Health Organization established billing codes with the production of the International Classification of Diseases. At the same time, biomedical researchers began to define data domains to capture and exchange. This began with the Gene Ontology Consortium capturing gene and gene product functional information as a mechanism to unify the representation of gene and gene product attributes across species in the Model Organism Databases.

**Figure 1:**
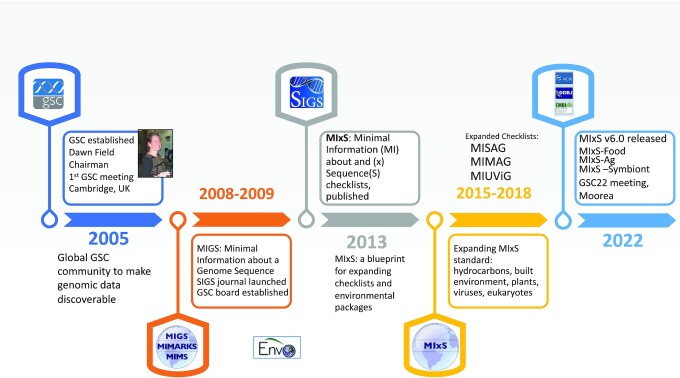
Timeline of standards development in the Genomic Standards Consortium (GSC).

In order to promote widespread access to omics data, the microarray research community established the first minimal information data standard for microarray experiments [1]. This was soon followed by other omic minimal information standards, including the Genomic Standard Consortium's Minimal Information for a Genome Sequence (MIGS) to a suite of genomic checklists (genome, metagenome, marker sequence, uncultured viruses, and single-cell genomes) and 20 MIxS environmental packages, which include the new MIxS-SA (Symbiont), MIxS-Ag (Agriculture MicrobMIGS), MIMS, and MIxS genomic metadata standards [2]. The first decade of the Genomic Standards Consortium (GSC) focused on the development and implementation of the GSC MIxS standards establishing the GSC as a standards body and developing consortium project collaborations [3,4]. This effort has brought together investigators working in different systems to work on a common problem. The GSC launched the *Standards in Genomic Sciences* (*SIGS*) journal in 2009 with the publication of *SIGS* transferring to BioMed Central as the *Environmental Microbiome Journal* (standardsingenomics.biomedcentral.com) in 2014 (Figure 1. —GSC milestones).

### The new normal—data standardization

The raison d'être of standards development has not abated in the past decade; rather, formalization of data with standards has become the “standard practice” for supporting FAIR data. This has driven development of large-scale data coordination efforts, enabling previously disparate resources to be connected via cross-standard mappings (e.g., Common Fund projects), as well as providing critical infrastructure for coordinating data to address evolving data needs during the COVID-19 pandemic (e.g., PHA4GE SARS-CoV-2 contextual data specification) [5].

The Open Biological and Biomedical Ontologies Foundry's establishment of common principles for development, sharing, and reuse of biomedical terms demonstrates the efforts of communities tackling a common problem [6]. These early efforts, such as anatomy terms by the Foundation Model of Anatomy, phenotypes by Phenotype and Trait Ontology, diseases in the Human Disease Ontology, and chemicals in Chemical Entities of Biological Interest, established broadly used resources. In the past decade, the breadth and impact of standards has expanded to include 966 ontologies accessible in the BioPortal (https://bioportal.bioontology.org) repository of biomedical ontologies, 182 interoperable biomedical domains and project-specific ontologies in the Open Biological and Biomedical Ontologies Foundry (https://obofoundry.org), and 37 minimal information standards (in FAIRsharing) [7]. The significant growth of data standards, as seen in FAIRsharing.org, has facilitated the expansion of awareness and utilization of interrelated data, metadata standards, databases, and data policies.

The MIxS genomic metadata standards, available in the GSC's GitHub repository (https://github.com/GenomicsStandardsConsortium/mixs), have expanded to address the growing breadth of genomics studies from the initial MIGS to a suite of genomic checklists (genome, metagenome, marker sequence, uncultured viruses, and single-cell genomes) and 20 MIxS environmental packages, which include the new MIxS-SA (Symbiont), MIxS-Ag (Agriculture Microbiome), and MIxS-Food (developed with the Food and Drug Administration's Center for Food Safety and Applied Nutrition) for the MIxS v6.0 released in March 2022.

The MIxS standard provides a benchmark against which it is possible to start bringing existing data sets together, to enable integration and to allow comparative analysis across previously noncomparable data types. The impact on the field of microbial ecology has been especially profound, essentially by providing a common language in which investigators could describe their project with its goals, hypotheses, and the sampling, processing, and sequencing approaches used to generate data. Additionally, it has provided extensive lists of contextual data that describe the environment in which the microbial assemblages were isolated. This common framework provides a foundation on which to build massive collaborative projects, such as the Earth Microbiome Project (EMP) [8], which uses these standards to capture and describe the microbial assemblages in tens of thousands of environmental samples. EMP sample metadata in the QIITA database can be searched using Redbiom, a cache service for sample metadata and data [9] (https://github.com/biocore/redbiom). As all these data are described in a common format, it is possible for any researcher to download data and analyze them in their preferred program with minimal difficulty. This vastly improves the previous model where researchers had to spend months trawling through the literature to bring together all appropriate data to test their hypotheses. The standards infrastructure provided by the GSC has enabled the vision of microbial ecology databases to expand; thus, we are now able to think bigger and test hypotheses with more statistical power than ever before.

Setting standards continues to be challenged by the “field of dreams” dilemma, as in “if we create it, will they come?” Essentially, will researchers find the standards useful beyond the initial use case they were developed for? For this, communication, outreach, and education are critical components to achieve broad utility for any standard. Collaborating with journals and data repositories is essential for expanding beyond the community that built the standard. Journals promote standards efforts through the publication of article series that highlight this work, such as the GSC series [10] that launched with the *GigaScience* journal in 2012. This was quickly followed with the publication of 244 GSC-related papers so far and the Microbiology of the Built Environment series in 2015 and 2017. The establishment of data standards policies and best practices moves the standards effort forward, from the new National Institutes of Health's Policy on Data Management and Sharing (https://grants.nih.gov/grants/policy/data_sharing) to data reporting policies that promote the use of standards across journals (e.g., *GigaScience, Scientific Data, Microbiome, Environmental Microbiome*, and *ISME*) and data repositories (e.g., ENA, BioSamples, NCBI's GenBank, BioSample, and Sequence Read Archive), which all require that your project metadata follow one of the community-derived or repository standards.

## Conclusion

The “Standards” movement has spurred the development of over 200 novel tools for querying and extracting standardized data, for example, from PubMed and the Sequence Read Archive, and of bespoke metadata schemas providing blueprints for reporting data, and it continues to evolve.

The impact of standards development is clearly evident when querying PubMed for “ontology” returns 37,410 results and when examining Google Scholar citations identifies 1,235 citations to the GSC's initial MIGS paper, 545 citations to the 2011 MIxS paper, 845 citations to the Human Disease Ontology 2012 paper, and 32,647 citations for the first gene ontology paper published in 2000.

From the first meeting in 2005, when GSC Chairman Dawn Field (2005–2014) established the consortium, the GSC's mission has been to build a community of researchers committed to creating and implementing genomic metadata standards to make data discoverable and reusable. Dawn Field (1969–2020), GSC founder and a pioneer in the field of genomic standards, is being honored at the GSC's annual meeting in Moorea, French Polynesia (March 2022), with the establishment of the “Dawn Field Award for Outstanding Contributions to Genomic Standards,” awarded this year to Raïssa Meyer at the Alfred Wegener Institute for her work on the Omic Biodiversity Observation Network (Omic BON).

### Editors' note

This commentary is part of a series to celebrate a Decade of *GigaScience*, to coincide with the 10th anniversary of our launch in July 2012. These papers take a look back at 10 years of advances in large-scale research as open science has become mainstream.

### Abbreviations

EMP: Earth Microbiome Project; GSC: Genomic Standards Consortium; MIGS: Minimal Information for a Genome Sequence.

### Data Availability

Not applicable.

### Competing Interests

Not applicable
